# Platelets and Neutrophil Apoptosis: A New Frontier in Inflammation Resolution

**DOI:** 10.7759/cureus.89916

**Published:** 2025-08-12

**Authors:** Aliu O Olatunji, Malik Sarsour, Leah Wuescher, Randall Worth

**Affiliations:** 1 Medical Microbiology and Immunology, University of Toledo, Toledo, USA

**Keywords:** apoptosis, immune modulation, inflammation resolution, neutrophils, platelets

## Abstract

Platelets are traditionally recognized for their role in hemostasis and thrombosis. Recent advances reveal their critical involvement in immune responses and inflammation. This review explores platelet biology, including activation mechanisms, release of bioactive molecules, interactions with immune cells, particularly neutrophils, and their regulation of inflammation resolution. We also discuss neutrophil apoptosis pathways and how platelet-derived factors influence this process. Understanding these interactions offers new insights into immune modulation and potential therapeutic targets for inflammatory diseases.

## Introduction and background

Overview of platelets

Their Traditional Role in Hemostasis and Thrombosis

Platelets, also known as thrombocytes, are small, anucleate cell fragments derived from megakaryocytes in the bone marrow. With a lifespan of approximately 7-10 days in circulation, platelets play a central role in maintaining vascular integrity and preventing blood loss through hemostatic and thrombotic processes [1]. Traditionally, platelets are understood primarily in the context of their contribution to hemostasis, where they act as first responders to vascular injury by forming a temporary plug at the site of damage and initiating the coagulation cascade. Their role in thrombosis, while similar in mechanism, involves pathological clot formation that can lead to vascular occlusion and ischemia [2].

Platelet Formation and Activation

Platelets are produced in the bone marrow through a process known as thrombopoiesis, in which large, polyploid megakaryocytes extend proplatelet extensions into sinusoidal blood vessels, releasing platelets into the circulation [3]. Upon injury to the vascular endothelium, platelets are rapidly activated by exposure to agonists, which bind to platelet receptors and initiate intracellular signaling cascades [4]. Activated platelets undergo several transformations important to their function. Platelets transition from a discoid to a spiky shape, increasing surface area and promoting adhesion. They release granule contents, which amplify the activation response and recruit additional platelets. Activation exposes surface receptors enabling platelet-platelet interactions that facilitate clot formation [5].

Hemostasis: Platelet Plug Formation and Coagulation Cascade

Hemostasis is the physiological process that prevents excessive bleeding following vascular injury. It consists of primary and secondary hemostasis [6]. Primary hemostasis involves the formation of a temporary "platelet plug." Platelets adhere to exposed collagen and von Willebrand factor (vWF) at the site of endothelial damage, becoming activated and recruiting additional platelets to form an aggregate. This plug is crucial for stemming blood loss in the initial stages post-injury [6]. Secondary hemostasis entails the activation of the coagulation cascade, which stabilizes the platelet plug by forming an insoluble fibrin mesh. This process involves the sequential activation of clotting factors, ultimately converting fibrinogen to fibrin, which cross-links and solidifies the clot [6]. Activated platelets also provide a phospholipid surface that accelerates the conversion of prothrombin to thrombin, a key step in fibrin formation [7]. Together, these processes ensure efficient blood clotting, with platelets at the center of initiating, sustaining, and stabilizing the clot.

Thrombosis: Pathological Clot Formation

While hemostasis is protective, thrombosis represents its pathological counterpart, where inappropriate clot formation occurs within intact blood vessels. Platelet-driven thrombosis is a key contributor to cardiovascular diseases such as myocardial infarction, stroke, and deep vein thrombosis. The process shares many features with hemostasis but occurs in the absence of overt vascular injury and is often associated with factors such as endothelial dysfunction, hypercoagulability, or abnormal blood flow [8]. In arterial thrombosis, high shear stress and disturbed blood flow expose vWF and collagen, triggering platelet adhesion and activation. The resulting platelet aggregates block blood flow and can cause tissue ischemia. In contrast, venous thrombosis is largely fibrin-driven, with platelets playing a secondary but supportive role in stabilizing the fibrin-rich clot [8-12].

## Review

Mechanisms of platelet activation and release of bioactive molecules

Platelet activation is a complex, highly regulated process essential to hemostasis and the inflammatory response. Upon vascular injury, platelets become activated, releasing a plethora of bioactive molecules stored within their granules, which play critical roles in blood clot formation, immune modulation, and tissue repair. The process of platelet activation and the subsequent release of granule-stored bioactive molecules involve several key signaling pathways and receptors. The intricate mechanisms underlying platelet activation not only govern hemostasis but also facilitate cross-talk with immune cells, modulating inflammation and immune responses [9,10].

Platelet activation pathways

Platelets are activated by a variety of stimuli, including contact with subendothelial matrix proteins, exposure to soluble agonists (such as thrombin and adenosine diphosphate (ADP)), and interaction with immune cell-derived molecules. When endothelial damage occurs, subendothelial collagen is exposed, allowing circulating platelets to adhere via glycoprotein VI (GPVI) and integrin α2β1 [5]. Upon adhesion, platelets undergo rapid shape change from discoid to a spread form, facilitating contact with other platelets and the exposed vessel wall. Another method of activation is when soluble agonists such as thrombin, ADP, and thromboxane A2 (TxA2) bind to specific platelet receptors (e.g., protease-activated receptors (PARs) for thrombin, P2Y12 for ADP, and thromboxane receptors) [8]. These interactions initiate intracellular signaling cascades that lead to further platelet activation and aggregation. One major result of platelet activation is an increase in intracellular calcium levels, which serves as a secondary messenger to activate various enzymes and signaling pathways, leading to the fusion of granules with the plasma membrane and release of bioactive molecules. Calcium mobilization is crucial for both the secretion of granule contents and the activation of integrins [4].

Immune-mediated activation of platelets

Beyond their classical roles in hemostasis and thrombosis, platelets have emerged as critical players in innate and adaptive immunity. They express a variety of immune receptors that allow them to sense and respond to pathogens, immune complexes, and inflammatory signals. These include pattern recognition receptors (PRRs) such as Toll-like receptors (TLRs), Fc gamma receptors (FcγRs), C-type lectin receptors (CLRs), and components of the inflammasome complex. Through these receptors, platelets contribute to pathogen detection, cytokine release, leukocyte recruitment, and immune amplification [10].

Platelets express several TLRs, including TLR1, TLR2, TLR3, TLR4, TLR5, TLR6, TLR7, and TLR9. These receptors recognize pathogen-associated molecular patterns (PAMPs) such as lipopolysaccharides (LPS), bacterial lipopeptides, viral RNA, and unmethylated CpG DNA. TLR4, for instance, is activated by LPS and promotes platelet aggregation, P-selectin (CD62P) expression, and cytokine release [11]. TLR2/1 and TLR2/6 recognize bacterial lipopeptides and induce NF-κB signaling. TLR7, which senses viral single-stranded RNA, mediates IL-1β production and platelet-neutrophil interactions during viral infections [12,13]. TLR9 is localized intracellularly and recognizes CpG motifs from bacteria and damaged host cells.

Human platelets express the low-affinity receptor FcγRIIA (CD32a), which binds the Fc portion of IgG. This receptor enables platelets to respond to immune complexes, playing roles in infection, autoimmunity, and drug-induced thrombocytopenia. In heparin-induced thrombocytopenia (HIT), PF4-heparin-IgG complexes activate platelets via FcγRIIA, leading to aggregation and thrombus formation [14]. FcγRIIA engagement results in ITAM signaling, granule release, and promotion of neutrophil extracellular trap (NET) formation.

The C-type lectin receptor (CLR) CLEC-2 is a prominent CLR expressed by platelets. It binds podoplanin and certain microbial ligands, initiating Syk-dependent signaling cascades. CLEC-2 is essential for maintaining vascular integrity and contributes to thromboinflammatory responses during infection [15].

Although platelets are anucleate, they contain components of the inflammasome such as NLRP3, caspase-1, and IL-1β. NLRP3 activation in platelets promotes the maturation and release of IL-1β, supporting leukocyte recruitment and vascular inflammation [16]. These inflammasome components allow platelets to function as immune effectors despite their limited transcriptional capacity (Table 1).

**Table 1:** Platelet immune receptors and their ligands and major functional outcomes LPS: lipopolysaccharide; NET: neutrophil extracellular trap; PAMPs: pathogen-associated molecular patterns; DAMPs: damage-associated molecular patterns

Receptor	Ligand(s)	Function	Reference
TLR2/1 and TLR2/6	Bacterial lipopeptides	NF-κB activation, inflammatory cytokine release	[13]
TLR4	LPS	Platelet activation, CD62P expression, cytokine secretion	[13]
TLR7	Single-stranded viral RNA	IL-1β production, platelet-neutrophil interaction	[13]
TLR9	CpG DNA (bacterial, mitochondrial origin)	Sensing of DNA, pro-inflammatory responses	[13]
FcγRIIA (CD32a)	Immune complexes (IgG bound to antigen)	Platelet aggregation, thromboinflammation, NET formation	[14]
CLEC-2	Podoplanin, microbial ligands	Maintenance of vascular integrity, thromboinflammatory response	[15]
NLRP3 inflammasome	PAMPs/DAMPs (cellular stress, infection)	IL-1β maturation and release, promotion of inflammation	[16-21]

Mechanisms of granule secretion and bioactive molecule release

The fusion of platelet granules with the plasma membrane is a tightly regulated process mediated by soluble N-ethylmaleimide-sensitive factor attachment protein receptor (SNARE) proteins. Upon activation, SNARE complexes facilitate granule fusion and the release of granule-stored molecules. SNARE proteins, including VAMP-3, SNAP-23, and syntaxin-4, are essential for the fusion of granules with the plasma membrane. Calcium signaling through calmodulin and protein kinases facilitates the assembly of SNARE complexes, promoting granule secretion [17]. Platelet activation also involves actin cytoskeleton rearrangement, which supports granule movement toward the plasma membrane. Actin and myosin interactions, regulated by Rho GTPases, are essential for efficient granule secretion [18].

Released bioactive molecules and their functions

The bioactive molecules released by platelets contribute not only to hemostasis but also to immune modulation and tissue repair. Fibrinogen, vWF, and thrombospondin facilitate platelet aggregation and thrombus formation, stabilizing the hemostatic plug [19]. Cytokines and chemokines such as IL-1β, CXCL4, and RANTES (regulated on activation, normal T cell expressed and secreted) attract leukocytes to injury sites, promoting inflammation and initiating immune responses. Growth factors like vascular endothelial growth factor (VEGF), platelet-derived growth factor (PDGF), and transforming growth factor-β (TGF-β) are involved in tissue repair, angiogenesis, and modulating the local immune environment [10]. 

Composition of platelet releasates: proteins, cytokines, chemokines, and growth factors

Upon activation, platelets release a broad array of bioactive molecules from their α-granules, dense granules, and lysosomes. These constituents are collectively termed "platelet releasate." The substances composing platelet releasates include a variety of proteins, cytokines, chemokines, and growth factors stored in granules that can become rapidly mobilized and secreted in response to platelet activation or can be delayed based on synthesis and storage (IL-1b and others) in response to activation. The release of these bioactive molecules occurs upon platelet activation through various stimuli, playing a crucial role in modulating inflammatory responses, immune modulation, tissue repair, and wound healing [20]. Alpha granules contain growth factors, clotting factors, and adhesion molecules, while dense granules contain smaller signaling molecules like ADP and calcium. These molecules not only recruit and activate additional platelets but also contribute to inflammation and vascular healing, highlighting the multifunctional role of platelets in hemostasis [21].

Upon activation, platelets release a broad array of bioactive molecules from their α-granules, dense granules, and lysosomes. These constituents, collectively termed the "platelet releasate," include proteins, small molecules, and other subcellular elements that modulate innate and adaptive immunity.

Platelet-derived cytokines, such as IL-1β, IL-6, TNF-α, and TGF-β1, are crucial modulators of the immune response, with roles ranging from immune cell recruitment to anti-inflammatory signaling [6]. Chemokines, including PF4 (CXCL4), RANTES (CCL5), MIP-1α, and SDF-1 (CXCL12), facilitate leukocyte chemotaxis and T-cell activation [22].

In addition, growth factors like PDGF, VEGF, epidermal growth factor (EGF), basic fibroblast growth factor (bFGF), IGF-1, and hepatocyte growth factor (HGF) promote angiogenesis, tissue regeneration, and cellular proliferation [23]. Platelets also contribute to coagulation through the release of clotting factors, such as fibrinogen, factor V, vWF, and plasminogen activator inhibitor-1 (PAI-1), which support thrombus formation and stability [24].

Antimicrobial peptides like thrombocidins and β-defensins are secreted in response to pathogens and contribute to direct microbial killing [25]. Adhesion molecules such as P-selectin, CD40L, and integrins (GPIIb/IIIa, GPIb) mediate platelet-leukocyte and platelet-endothelial interactions [26]. In parallel, matrix and extracellular matrix (ECM) proteins, including osteonectin and thrombospondin-1, play roles in wound healing and tissue remodeling [27].

Small molecules are also prominent in the platelet releasate. Amines like serotonin and histamine influence vasoconstriction and immune cell behavior [28]. Platelets release nucleotides (ADP, adenosine triphosphate (ATP), adenosine monophosphate (AMP), cyclic adenosine monophosphate (cAMP)), which act through purinergic signaling to promote activation and aggregation [29]. Polyamines such as spermidine and spermine have been implicated in oxidative stress regulation and apoptosis modulation [30]. Furthermore, bioactive lipids, including platelet-activating factor (PAF), thromboxane A2, and prostaglandins, play essential roles in inflammation and vascular tone [31].

The other bioactive fractions of platelet releasates include microparticles (PMVs), microRNAs, functional mitochondria, and reactive oxygen species (ROS). Platelet-derived microparticles carry RNA, proteins, and lipids and are instrumental in cell-cell communication during inflammation and thrombosis [32]. Platelet-enriched microRNAs (e.g., miR-223, miR-21, miR-126) have gene regulatory functions in endothelial and immune cells [33]. Mitochondria released by activated platelets are biologically functional and modulate innate immunity and inflammatory responses [34]. Lastly, ROS, including hydrogen peroxide and superoxide, contribute to microbial killing and immune signaling [35] (Table 2). Collectively, these molecules reflect the multifaceted role of platelets as immune cells, influencing immunity through chemotaxis, cytokine release, antimicrobial activity, and intercellular communication. 

**Table 2:** Components of platelet releasates RANTES: regulated on activation, normal T cell expressed and secreted; PDGF: platelet-derived growth factor; VEGF: vascular endothelial growth factor; EGF: epidermal growth factor; bFGF: basic fibroblast growth factor; HGF: hepatocyte growth factor; vWF: von Willebrand factor; ADP: adenosine diphosphate; ATP: adenosine triphosphate; AMP: adenosine monophosphate; cAMP: cyclic adenosine monophosphate; PAF: platelet-activating factor; ROS: reactive oxygen species

Category	Subcategory	Examples	Function/type	Key references
Proteins	Cytokines	IL-1β, IL-6, TNF-α, TGF-β1	Pro-/anti-inflammatory mediators; immune modulation	[22]
	Chemokines	PF4 (CXCL4), RANTES (CCL5), MIP-1α, SDF-1 (CXCL12)	Leukocyte recruitment, T-cell and neutrophil activation	[22]
	Growth factors	PDGF, VEGF, EGF, bFGF, IGF-1, HGF	Angiogenesis, tissue repair, cell proliferation	[23]
	Clotting factors	Fibrinogen, factor V, vWF, PAI-1	Coagulation cascade, platelet aggregation	[24]
	Antimicrobial peptides	Thrombocidins, kinocidins, β-defensins	Host defense, bacterial killing	[25]
	Adhesion molecules	P-selectin, CD40L, GPIIb/IIIa, GPIb	Platelet-endothelial/leukocyte interactions	[26]
	Matrix/ECM-related proteins	Osteonectin, thrombospondin-1, fibronectin	Matrix remodeling, wound healing	[27]
Small molecules	Amines	Serotonin (5-HT), histamine	Vasoconstriction, mood regulation, immune modulation	[28]
	Nucleotides	ADP, ATP, AMP, cAMP	Platelet activation, purinergic signaling	[29]
	Polyamines	Spermidine, spermine	ROS scavenging, apoptosis modulation	[30]
	Lipids	PAF, thromboxane A2, prostaglandins	Inflammation, vasoconstriction, pain	[31]
Other factors	Microparticles (PMVs)	Platelet-derived microvesicles	Carry proteins, RNA, lipids to modulate immunity and thrombosis	[32]
	MicroRNAs	miR-223, miR-21, miR-126	Regulate gene expression in immune and endothelial cells	[33]
	Mitochondria	Functional mitochondria released during activation	Modulate inflammation, immunity	[34]
	ROS	H2O2, superoxide	Signaling and microbial killing	[35]

Emerging roles of platelets in immune modulation and inflammation

Beyond their traditional role in hemostasis and thrombosis, platelets have emerged as key players in immune modulation and inflammation. They interact closely with immune cells, secrete a wide array of bioactive molecules, and participate in immune signaling, bridging the gap between innate immunity and coagulation. This expanding role is particularly relevant in conditions such as infection, chronic inflammation, and autoimmune diseases, where platelets contribute to host defense mechanisms, pathogen clearance, and immune regulation [36].

Platelet-immune cell interactions

Platelets express various surface molecules that enable them to interact directly with immune cells, such as neutrophils, monocytes, and lymphocytes. Through these interactions, platelets can influence immune cell function and direct immune responses. Platelets adhere to neutrophils primarily via P-selectin, which binds to P-selectin glycoprotein ligand-1 (PSGL-1) on neutrophils [37]. This interaction promotes the formation of NETs, which help trap and neutralize pathogens but can also contribute to thrombosis and tissue damage in diseases like COVID-19 and sepsis [38]. Platelets activate monocytes by releasing pro-inflammatory cytokines and chemokines, such as IL-1β and RANTES. This activation can lead to monocyte differentiation and the promotion of a pro-inflammatory phenotype that accelerates atherosclerosis and other chronic inflammatory conditions [39]. Platelets modulate T and B lymphocytes, influencing adaptive immunity. They express major histocompatibility complex (MHC) class I molecules and present antigens to T cells, indirectly contributing to adaptive immune responses. Platelets also release factors that modulate T-cell activation and proliferation, suggesting a role both in protective immunity and in conditions where immune tolerance is disrupted [40]. Platelets can activate peripheral blood B cells and increase the production of immunoglobulins through the expression of CD40L [41].

Platelet activation in infection and sepsis

Platelets serve as frontline responders in immune defense, particularly during bacterial and viral infections. During infection, platelets interact with pathogens directly and enhance the immune response. Platelets express pathogen recognition receptors (PRRs) such as TLRs, allowing them to directly sense microbial components. For example, TLR4 on platelets recognizes LPS from Gram-negative bacteria, leading to platelet activation and immune signaling. This activation supports pathogen clearance through platelet-leukocyte aggregation and enhances the immune response to the pathogen [42]. In sepsis, platelets release pro-inflammatory cytokines and chemokines that amplify systemic inflammation, which is a hallmark of septic shock. Activated platelets in sepsis can also form aggregates with leukocytes, contributing to microvascular thrombosis and organ dysfunction. This dual role underscores the complex involvement of platelets in balancing pathogen defense and exacerbating inflammatory damage [43].

Platelet-driven inflammatory diseases

Platelets have been implicated in the pathogenesis of various inflammatory and autoimmune diseases through their interactions with immune cells and release of inflammatory mediators. In atherosclerosis, platelets contribute to plaque formation and progression through their interactions with endothelial cells and monocytes. Activated platelets release pro-inflammatory cytokines, chemokines, and growth factors that promote monocyte adhesion, foam cell formation, and smooth muscle cell migration, all key steps in atherogenesis [44]. In autoimmune diseases such as systemic lupus erythematosus (SLE) and rheumatoid arthritis, platelets exacerbate inflammation through their release of pro-inflammatory cytokines and promotion of immune cell recruitment. In SLE, platelets contribute to immune complex formation and NET formation, leading to further inflammation and tissue damage [45]. Platelets support tumor growth and metastasis through their ability to shield circulating tumor cells from immune detection, promote angiogenesis, and release factors that facilitate tissue invasion and metastasis. Tumor-educated platelets can even alter their RNA profiles to support pro-metastatic pathways, showing how platelets can adapt to and promote tumor progression [46].

Platelets and immune tolerance

In addition to their pro-inflammatory roles, platelets also promote immune tolerance under certain conditions. Platelet-derived TGF-β and other anti-inflammatory cytokines help maintain immune homeostasis and prevent excessive immune activation. This immune-suppressive capacity can be beneficial in chronic inflammatory diseases and autoimmune conditions, where platelets may modulate excessive immune responses [47]. The role of platelets in immune modulation and inflammation is complex and multifaceted, encompassing both pro- and anti-inflammatory functions. Platelets not only support hemostasis but also interact with various immune cells, release a broad spectrum of cytokines and chemokines, and respond directly to pathogens.

Effects of platelets on immune cells

Platelet releasates are important not only for hemostasis but also for modulating immune responses and inflammation. Upon activation, platelets release these molecules from their granules, influencing various immune cells, including neutrophils, monocytes, dendritic cells, and lymphocytes. This broad modulatory capability situates platelets as central players in the immune system, bridging innate and adaptive responses, amplifying inflammation, and orchestrating tissue repair and regeneration [48]. Below is a detailed overview of how platelets interact with immune cells and modulate inflammatory processes.

Platelet-neutrophil interaction

Platelet-neutrophil interactions are pivotal in inflammation, especially in the early stages. Neutrophils are rapidly recruited to sites of infection or injury and are often the first immune cells to interact with activated platelets. Platelet-derived chemokines such as CXCL4 (platelet factor 4) and CXCL7 attract neutrophils to sites of inflammation, enhancing neutrophil migration and activation [49]. Additionally, platelet-neutrophil complexes increase during inflammation, promoting neutrophil activation and the release of ROS, which aid in pathogen elimination. Platelet releasates containing molecules like IL-1β and CD40L enhance NET formation, a mechanism in which neutrophils release web-like structures composed of DNA, histones, and antimicrobial proteins to trap and kill pathogens. This platelet-driven NET formation has been shown to be particularly critical in infections, such as sepsis [50].

Effects on monocytes and macrophages

Platelet releasates also profoundly impact monocytes and macrophages, both of which play major roles in antigen presentation and inflammation resolution. Through the release of monocyte chemoattractant proteins (MCP-1/CCL2), platelet releasates recruit monocytes to inflammatory sites, where they differentiate into macrophages or dendritic cells, amplifying local immune responses [51]. Additionally, platelet-derived CD40L enhances monocyte activation and cytokine production, thereby contributing to the inflammatory cascade [52]. Platelet-derived TGF-β and other cytokines influence macrophage polarization. Studies have shown that platelet releasates can skew macrophages toward an M2 phenotype, supporting tissue repair and inflammation resolution [53]. Conversely, in a pro-inflammatory environment, platelet-derived factors like CD40L and IL-1β can promote an M1 macrophage phenotype, associated with a heightened inflammatory response [53].

Effects on dendritic cells

Dendritic cells are critical antigen-presenting cells that bridge innate and adaptive immunity, and platelet-derived factors significantly impact their function. Platelet-derived CD40L has been shown to enhance dendritic cell maturation, a process essential for effective antigen presentation to T cells [54]. Studies indicate that platelet releasates also contain interleukins that promote dendritic cell activation and cytokine release, enhancing adaptive immune responses [55]. Platelet-derived molecules such as RANTES (CCL5) increase the recruitment and activation of DCs, promoting the development of pro-inflammatory T-helper cell responses [10]. This immunogenic influence of platelet releasates on dendritic cells underscores their role in adaptive immune activation, particularly in chronic inflammatory conditions and autoimmune diseases.

Effects on T and B lymphocytes

Platelet releasates affect both T and B lymphocytes, thus influencing adaptive immune responses. Platelet-derived CD40L and IL-1β are known to stimulate T-cell activation and proliferation. By interacting with T-cell CD40 and IL-1 receptors, platelet releasates facilitate the differentiation and expansion of T-helper cells, enhancing immune responses to pathogens or injury [40]. These interactions are especially relevant in chronic inflammatory diseases, where excessive T-cell activation by platelet releasates can exacerbate tissue damage. While the effects of platelet releasates on B cells are less well-studied, platelet-derived CD40L has been implicated in supporting B-cell proliferation and antibody production. This effect is critical for the development of humoral immunity and may also play a role in autoimmune conditions [56].

Amplification of inflammation

Beyond direct immune cell modulation, platelet releasates play a pivotal role in amplifying inflammatory responses at sites of tissue damage or infection. Platelet-derived factors like serotonin and TxA2 increase endothelial cell permeability and adhesion molecule expression, facilitating immune cell infiltration at sites of injury [22]. This endothelial activation is fundamental for immune cell extravasation and enhances inflammation. The release of pro-inflammatory cytokines such as IL-6 and TNF-α by platelets creates a feedback loop that sustains the inflammatory response. Platelets in inflamed tissue release these cytokines, which attract more immune cells and induce further cytokine release from resident cells, creating a persistent inflammatory microenvironment [57]. Growth factors released by platelets, such as VEGF and PDGF, promote angiogenesis and tissue repair, which are necessary for resolving inflammation. However, in pathological conditions, such as cancer, these factors can lead to aberrant angiogenesis, supporting tumor growth and metastasis [58].

Platelet releasates contain a diverse array of bioactive molecules that modulate immune cell functions and amplify inflammatory responses. From attracting neutrophils and monocytes to enhancing T-cell responses, platelet-derived factors orchestrate a wide range of immune activities. This versatility highlights the role of platelets not only in hemostasis but also as critical mediators of immune and inflammatory processes.

Intrinsic and extrinsic pathways of neutrophil apoptosis

Neutrophils, as short-lived immune effector cells, undergo apoptosis to prevent excessive tissue damage and inflammation. This tightly regulated cell death is essential to maintaining immune balance and is controlled by two primary apoptotic pathways: the intrinsic (mitochondrial) pathway and the extrinsic (death receptor-mediated) pathway. Both pathways are crucial for neutrophil homeostasis, facilitating the timely removal of these cells through phagocytosis by macrophages [59].

Intrinsic Pathway

The intrinsic, or mitochondrial, pathway of apoptosis is primarily controlled by the B-cell lymphoma 2 (Bcl-2) family proteins. These proteins regulate mitochondrial membrane integrity and can either promote or inhibit apoptosis. Key pro-apoptotic members include Bax, Bak, and Bid, which promote mitochondrial outer membrane permeabilization (MOMP) in response to cellular stress, leading to the release of cytochrome c from mitochondria into the cytosol [60]. The Bcl-2 family contains both pro-apoptotic and anti-apoptotic proteins, forming a regulatory network within the mitochondria that determines cell fate. In neutrophils, proteins like Mcl-1 and Bcl-xL counterbalance the pro-apoptotic proteins to delay apoptosis. However, during inflammation or in response to cellular stress, pro-apoptotic proteins like Bax and Bak are activated, leading to MOMP [61]. This process is crucial in maintaining neutrophil survival under homeostatic conditions and triggering apoptosis under stress. Upon mitochondrial membrane permeabilization, cytochrome c is released into the cytosol, where it binds to apoptotic protease-activating factor-1 (Apaf-1). This binding leads to the formation of the apoptosome, a protein complex that recruits and activates initiator caspase-9. Caspase-9, in turn, activates downstream executioner caspases, such as caspase-3 and caspase-7, leading to characteristic apoptotic cell changes, including chromatin condensation, DNA fragmentation, and membrane blebbing [62]. ROS are by-products of neutrophil respiratory bursts during immune responses. These ROS can induce oxidative damage, promoting MOMP and the release of pro-apoptotic molecules. Elevated ROS levels within neutrophils have been linked to mitochondrial dysfunction and the activation of the intrinsic apoptotic pathway, especially after immune activation or during prolonged inflammation [63].

Extrinsic Pathway

The extrinsic pathway is initiated by the binding of extracellular death ligands to specific death receptors on the neutrophil surface. This pathway is particularly relevant in regulating immune responses, where cell-cell interactions often determine neutrophil lifespan. The primary receptors in the extrinsic pathway include Fas (CD95), TNF receptor 1 (TNFR1), and TNF-related apoptosis-inducing ligand (TRAIL) receptors (DR4 and DR5). These receptors are activated by their respective ligands, such as Fas ligand (FasL) and tumor necrosis factor-alpha (TNF-α), which are typically expressed by immune cells like T cells and macrophages in the context of inflammation [64]. The binding of these ligands to their receptors triggers receptor trimerization and the recruitment of adaptor proteins like Fas-associated death domain protein (FADD). Upon FADD recruitment, caspase-8 is activated, which then initiates the caspase cascade, leading to apoptosis. Caspase-8 can directly activate executioner caspases like caspase-3 or cleave Bid, a BH3-only protein, linking the extrinsic pathway to mitochondrial events of the intrinsic pathway [65]. This cross-talk between pathways amplifies the apoptotic signal, ensuring efficient neutrophil clearance. The TRAIL can also induce apoptosis in neutrophils through TRAIL receptors. This pathway is particularly significant in chronic inflammation and autoimmune diseases, where TRAIL-mediated neutrophil apoptosis can help regulate immune responses and prevent excessive inflammation [64].

Cross-Talk Between Pathways

Although the intrinsic and extrinsic pathways are distinct, they are interconnected in neutrophils. Caspase-8, which is activated by extrinsic signals, can cleave Bid, which then translocates to the mitochondria, promoting Bax/Bak-mediated cytochrome c release and activation of the intrinsic pathway. This cross-talk ensures that death receptor signals can fully engage apoptosis, even under conditions where mitochondrial integrity is relatively intact [66].

Regulation during inflammation

In an inflammation, the apoptosis of neutrophils is delayed to extend their lifespan and enhance their immune functions. Pro-inflammatory cytokines, such as IL-8, GM-CSF, and interferon-gamma (IFN-γ), inhibit both intrinsic and extrinsic apoptotic pathways in neutrophils, promoting prolonged survival at infection sites [67]. Conversely, resolution signals like TGF-β and IL-10 promote neutrophil apoptosis, facilitating the resolution of inflammation and the clearance of apoptotic neutrophils by macrophages [68].

Clinical relevance

Dysregulation of neutrophil apoptosis contributes to various diseases. Delayed apoptosis is observed in chronic inflammatory diseases like rheumatoid arthritis and chronic obstructive pulmonary disease (COPD), leading to neutrophil accumulation and tissue damage [69]. Conversely, enhanced neutrophil apoptosis can weaken host defense in infections, as seen in conditions with increased neutrophil turnover, such as sepsis [70].

The intrinsic and extrinsic pathways of apoptosis are essential for regulating neutrophil turnover, ensuring that these cells are removed after fulfilling their immune functions. This regulation is critical for maintaining tissue homeostasis and preventing immune-related pathology. Advances in understanding neutrophil apoptosis pathways hold potential for therapeutic strategies aimed at modulating neutrophil lifespan in inflammatory diseases and infections.

Role of apoptosis in resolving inflammation and preventing tissue damage

Apoptosis, or programmed cell death, is a controlled process that plays a vital role in maintaining tissue homeostasis and regulating immune responses. In the context of inflammation, apoptosis serves as a major mechanism for resolving inflammatory responses and limiting tissue damage. By removing immune cells that have fulfilled their roles, apoptosis helps to clear the site of infection or injury and promote tissue healing [71]. Apoptosis is particularly important in the resolution of inflammation because it prevents the release of intracellular contents that could otherwise exacerbate inflammatory processes and cause further damage [72].

Apoptosis in immune regulation

During an immune response, leukocytes, such as neutrophils and macrophages, are recruited to sites of infection or injury. These cells perform essential functions, including pathogen elimination and cytokine release, to orchestrate the immune response. However, prolonged activation and survival of these immune cells can lead to excessive inflammation and tissue damage [73]. Apoptosis serves to remove these cells in a controlled manner, allowing the immune system to shift from a pro-inflammatory to an anti-inflammatory state. Neutrophils are often the first responders in an immune response, and they undergo apoptosis once their antimicrobial functions are completed. Neutrophil apoptosis is essential for preventing prolonged inflammation and subsequent tissue damage. When neutrophils undergo apoptosis, they lose their pro-inflammatory potential, thereby reducing the release of damaging enzymes and ROS [64]. This process is particularly important in diseases characterized by chronic inflammation, such as COPD and rheumatoid arthritis, where delayed neutrophil apoptosis contributes to tissue destruction [74]. Apoptotic cells expose specific "eat me" signals, such as phosphatidylserine, on their surface, which are recognized by macrophages and other phagocytic cells. This recognition promotes the efficient clearance of apoptotic cells through a process known as efferocytosis [75]. Efferocytosis not only removes apoptotic cells but also triggers anti-inflammatory signaling pathways in macrophages, promoting the release of cytokines like IL-10 and TGF-β. These cytokines contribute to the resolution of inflammation and support tissue repair [76].

Prevention of tissue damage

One of the critical roles of apoptosis in inflammation resolution is preventing secondary necrosis, which occurs when apoptotic cells are not promptly cleared and instead undergo cell lysis. Unlike apoptosis, which is non-inflammatory, necrosis releases cellular contents, including damage-associated molecular patterns (DAMPs) such as HMGB1 and ATP, into the surrounding tissue. These DAMPs can trigger inflammatory pathways, recruit additional immune cells, and amplify tissue damage [77]. The prompt clearance of apoptotic cells by macrophages prevents secondary necrosis, thus reducing the likelihood of prolonged inflammation and further tissue injury [78]. Inefficient apoptosis or defective clearance of apoptotic cells is associated with autoimmune and chronic inflammatory diseases. For example, in SLE, defective apoptotic cell clearance leads to the accumulation of apoptotic debris, which can act as autoantigens and trigger immune responses against self-tissues [79]. In contrast, efficient apoptosis and efferocytosis help maintain immune tolerance by clearing potentially antigenic cellular material before it can induce an immune response [80].

Apoptosis and the shift toward tissue repair and resolution

The resolution of inflammation involves a transition from a pro-inflammatory to a pro-resolving environment, a shift that is heavily influenced by the apoptosis of immune cells. After clearing apoptotic cells, macrophages undergo a phenotypic change, shifting from a pro-inflammatory (M1) to a pro-resolving (M2) phenotype. This change is critical for the release of pro-resolving mediators and growth factors, such as VEGF and HGF, which support tissue regeneration and repair [81]. Specialized pro-resolving lipid mediators, such as resolvins, protectins, and lipoxins, are released during the resolution phase and enhance the apoptosis of neutrophils. These mediators not only promote apoptosis but also facilitate efferocytosis and tissue repair, helping to restore tissue integrity and function after inflammation [82]. Lipoxin A4, for instance, enhances neutrophil apoptosis and clearance by macrophages, thus supporting the resolution of inflammation [83]. Apoptosis of fibroblasts and other stromal cells also plays a role in tissue remodeling during the resolution phase of inflammation. After inflammatory cells are cleared, apoptosis of activated fibroblasts prevents excessive scarring and fibrosis, promoting functional tissue repair rather than pathological tissue remodeling [84].

Clinical implications

The understanding of apoptosis as a key mechanism in resolving inflammation has important therapeutic implications. Strategies aimed at inducing apoptosis in overactive immune cells, or enhancing efferocytosis, have potential applications in treating chronic inflammatory and autoimmune diseases. Drugs that mimic the effects of pro-resolving lipid mediators, for example, are being explored for their ability to promote inflammation resolution without compromising immune defenses [82]. In chronic inflammatory conditions, targeting apoptotic pathways to enhance the clearance of inflammatory cells has shown promise. In rheumatoid arthritis, for instance, therapies that increase the apoptosis of neutrophils and macrophages at inflammation sites can reduce joint damage and improve patient outcomes [85].

Apoptosis is essential for the resolution of inflammation and the prevention of tissue damage. By regulating the timely removal of immune cells from inflammatory sites, apoptosis prevents excessive immune responses and supports the transition to tissue healing and repair. Impaired apoptosis and clearance of apoptotic cells are associated with chronic inflammation and autoimmune diseases, highlighting the importance of apoptosis in immune regulation and tissue homeostasis. Advances in understanding the role of apoptosis in inflammation resolution offer promising therapeutic strategies for managing chronic inflammatory diseases.

Influence of external factors on neutrophil apoptosis

Neutrophil apoptosis is a major event in the resolution of inflammation, as it ensures the removal of these cells after they have performed their antimicrobial functions. The regulation of neutrophil apoptosis is influenced by a variety of external factors, including cytokines, chemokines, growth factors, and other soluble molecules, many of which are derived from platelets [72]. Platelets, traditionally known for their role in hemostasis, have increasingly been recognized for their involvement in immune modulation and inflammation resolution. Through the release of bioactive molecules, platelets can influence neutrophil survival, migration, and apoptosis [86]. This section discusses how platelet-derived molecules and other external factors affect neutrophil apoptosis, with an emphasis on the mechanisms through which platelets modulate neutrophil fate during inflammatory responses.

Platelet-derived molecules and their role in neutrophil apoptosis

Platelets release a broad range of molecules that can directly influence neutrophil apoptosis, including cytokines, chemokines, growth factors, and extracellular vesicles. These factors can promote neutrophil survival in the early stages of inflammation or induce apoptosis to resolve the inflammatory response once the threat has been cleared [87].

Cytokines, chemokines, and growth factors

Several platelet-derived cytokines and chemokines influence neutrophil apoptosis by modulating signaling pathways that control cell survival. Among the most well-characterized platelet-derived mediators involved in neutrophil apoptosis are IL-1β, TNF-α, and TGF-β [22]. Platelet-derived TNF-α has been shown to regulate neutrophil apoptosis through the extrinsic pathway [88]. TNF-α binds to its receptors (TNFR1 and TNFR2) on neutrophils, activating caspase-8, which can initiate apoptosis [88]. TNF-α can also induce the release of ROS in neutrophils, which further promotes apoptotic signaling [89]. The IL-1β, another pro-inflammatory cytokine released by platelets, can both promote neutrophil activation and inhibit apoptosis, especially during the early stages of inflammation [90]. In contrast to TNF-α, IL-1β prolongs neutrophil survival through the activation of survival pathways like NF-κB and MAPK [90,91]. However, when the inflammatory signal subsides, the presence of IL-1β can shift, leading to enhanced apoptosis as part of the resolution process [92]. Platelets are also a major source of TGF-β, a cytokine known for its role in immune regulation. TGF-β has a complex role in neutrophil apoptosis. In some contexts, TGF-β promotes neutrophil survival, while in other circumstances, it enhances their clearance through apoptosis [93]. The effect of TGF-β on neutrophil apoptosis is largely dependent on the local microenvironment and the timing of its release [94].

Platelets also release a range of growth factors that can modulate neutrophil survival, migration, and apoptosis. These include VEGF and PDGF, both of which are implicated in inflammation and immune modulation. VEGF, released by activated platelets, can support the survival of neutrophils in inflamed tissues by promoting the formation of new blood vessels and enhancing the ability of neutrophils to reach the site of infection [95]. Additionally, VEGF has been shown to delay apoptosis in neutrophils by upregulating anti-apoptotic proteins such as Bcl-2 [96]. However, when the inflammatory stimulus resolves, the reduction of VEGF levels can shift the neutrophil response toward apoptosis. PDGF, which is released by platelets during inflammation, plays a role in neutrophil migration and activation. While PDGF is known for its pro-inflammatory effects, it can also influence neutrophil apoptosis by activating signaling pathways that promote cell survival, such as PI3K/Akt [97]. The sustained presence of PDGF in tissues can therefore prolong neutrophil lifespan, delaying apoptosis until the inflammatory stimulus is cleared.

Platelet-derived extracellular vesicles

Platelet-derived extracellular vesicles, including microparticles and exosomes, are another important mechanism by which platelets influence neutrophil apoptosis. These extracellular vesicles carry a variety of molecules such as microRNAs, cytokines, and enzymes that can affect neutrophil function and survival. Platelet-derived extracellular vesicles have been shown to contain microRNAs (miRNAs) that can influence gene expression in target cells, including neutrophils. For example, platelet-derived miR-223 has been implicated in neutrophil survival and migration by modulating the expression of pro-apoptotic and anti-apoptotic genes [98]. The transfer of miR-223 from platelets to neutrophils has been shown to enhance neutrophil effector functions and delay apoptosis in inflammatory conditions [99]. Extracellular vesicles derived from activated platelets also contain various cytokines and chemokines that can modulate neutrophil behavior. For example, platelet-derived extracellular vesicles enriched in IL-1β and TNF-α can prolong neutrophil survival, whereas those containing TGF-β can promote their apoptotic clearance after the resolution of inflammation [100].

Other environmental factors

In addition to platelet-derived molecules, other external factors, such as bacterial products, oxidative stress, and hypoxia, also play significant roles in modulating neutrophil apoptosis. During infection, bacterial components such as LPS and formylated peptides can delay neutrophil apoptosis, allowing these cells to remain active at the site of infection for extended periods [101]. This delay in apoptosis ensures that neutrophils can combat the invading pathogens effectively. However, persistent exposure to bacterial stimuli can eventually lead to excessive neutrophil activation and delayed apoptosis, contributing to chronic inflammation [102]. Oxidative stress, resulting from the generation of ROS, is a major factor influencing neutrophil survival and apoptosis. While ROS are crucial for neutrophil function during pathogen elimination, excessive ROS production can overwhelm the cell's antioxidant defenses, leading to cell damage and inducing apoptosis. Platelets, through the release of ROS-inducing molecules like TNF-α, can modulate this process by accelerating the activation of apoptotic signaling pathways [103]. Hypoxic conditions, which are common in inflamed tissues, can also affect neutrophil apoptosis. Hypoxia-inducible factors (HIFs), which are activated under low oxygen conditions, can modulate neutrophil survival. For example, HIF-1α has been shown to enhance neutrophil survival by upregulating anti-apoptotic molecules, thus extending the neutrophil lifespan during inflammation [104]. Influenza virus infection is characterized by an aggressive immune response aimed at clearing the virus from the respiratory tract. Neutrophils are among the first immune cells to respond, rapidly infiltrating the lungs and contributing to viral clearance through phagocytosis and the release of ROS and antimicrobial peptides [105]. Sendai virus, a murine parainfluenza virus, serves as a model for studying respiratory viral infections. Similar to influenza, Sendai virus infection leads to robust neutrophil recruitment to the lungs. Platelet involvement in Sendai virus infection is pivotal for both neutrophil recruitment and apoptosis [106].

Case studies in viral infections

Upon infection, platelets recognize viral components through TLRs, such as TLR3, TLR7, and TLR9. This recognition leads to platelet activation and degranulation, releasing chemokines and cytokines that attract neutrophils [107]. Additionally, platelets release pro-apoptotic factors like FasL and TRAIL, which bind to their respective receptors on neutrophils, triggering apoptosis [108]. This process ensures the removal of spent neutrophils, thereby resolving inflammation and limiting tissue damage.

In SARS-CoV-2 infection, activated platelets release a plethora of cytokines and chemokines that recruit neutrophils to the lungs. Platelets also express surface molecules like P-selectin, facilitating the formation of platelet-neutrophil aggregates [109]. These aggregates not only enhance neutrophil activation but also contribute to the formation of NETs, which can trap and neutralize the virus [110]. However, excessive NET formation can lead to lung tissue damage and contribute to the development of acute respiratory distress syndrome (ARDS) [111]. Moreover, platelet-derived soluble mediators, including FasL and TRAIL, induce neutrophil apoptosis, which is crucial for resolving inflammation. Dysregulation of this process can result in persistent inflammation and severe tissue damage, as observed in severe COVID-19 cases [112].

The regulation of neutrophil apoptosis is tightly controlled by a combination of intrinsic and extrinsic factors, including those derived from platelets. Platelet-derived molecules such as cytokines, chemokines, growth factors, and extracellular vesicles can either promote neutrophil survival in the early stages of inflammation or induce their apoptosis during the resolution phase. By modulating the timing and extent of neutrophil apoptosis, platelets play a crucial role in both promoting the effective response to infection and ensuring the resolution of inflammation. Further studies investigating the interplay between platelets, neutrophils, and other immune cells will provide deeper insights into the mechanisms of inflammation resolution and tissue repair, which could lead to the development of novel therapeutic strategies for inflammatory diseases.

## Conclusions

Platelets, traditionally recognized for their primary role in hemostasis, have now been established as a major component of the immune response, particularly during viral infections. This review has delved into the intricate mechanisms through which platelets influence neutrophil apoptosis, highlighting both their direct and indirect interactions. The multifaceted roles of platelets extend beyond clot formation, encompassing the modulation of immune responses and the resolution of inflammation.

Through direct interactions, platelets can influence neutrophil behavior by binding to them via surface receptors, releasing soluble mediators, and forming platelet-neutrophil aggregates. These interactions can promote or inhibit neutrophil apoptosis depending on the context of the infection and the local immune environment. Indirectly, platelets modulate the immune response by releasing a variety of cytokines and chemokines that affect neutrophil survival and function.

The mechanisms underlying platelet-neutrophil interactions during viral infections provide valuable insights into the resolution of inflammation and the overall immune response. This knowledge is critical for developing therapeutic strategies aimed at managing viral diseases. By targeting specific pathways through which platelets influence neutrophil apoptosis, it may be possible to enhance the clearance of viral infections while minimizing tissue damage and chronic inflammation.

In summary, platelets not only are essential for hemostasis but also play a pivotal role in orchestrating the immune response during viral infections. Their ability to regulate neutrophil apoptosis and modulate inflammation underscores their importance in both acute and chronic phases of viral diseases. Continued research in this area will undoubtedly yield novel therapeutic approaches, ultimately improving the management and outcome of viral infections.
